# Phospho-ERK levels as predictors for chemotherapy of rectal carcinoma

**DOI:** 10.18632/oncotarget.26741

**Published:** 2019-03-01

**Authors:** Susanne Holck, Louise Laurberg Klarskov, Lars-Inge Larsson

**Affiliations:** ^1^ Department of Pathology, Copenhagen University Hospital Hvidovre, DK-2650 Hvidovre, Denmark; ^2^ Department of Pathology, Copenhagen University Hospital Herlev, DK-2730 Herlev, Denmark; ^3^ Clinical Research Center, Copenhagen University Hospital Hvidovre, DK-2650 Hvidovre, Denmark

**Keywords:** rectal cancer, chemotherapy, fluorouracil, ERK, phosphorylation

## Abstract

Treatment of rectal cancer has been vastly improved by advances in surgery and radiochemotherapy but remains an important cause of morbidity and mortality worldwide. A particular problem is the lack of predictive markers that can help to individualize treatment. The growth- and apoptosis-regulating signaling molecules ERK 1 and 2 are important to cancer growth and progression. They are activated through phosphorylation, which is initiated by a cascade involving the EGF receptor and RAS as upstream regulators. Moreover, *in vitro* studies indicate that phospho-ERKs interfere with 5-fluorouracil-based chemotherapy. Recently, we showed that high levels of phospho-ERKs in rectal cancer cells predict poor responses to neoadjuvant (preoperative) radiochemotherapy. We now report that preoperative phospho-ERK levels also can subdivide high-risk rectal cancer patients into a favorable and a poor prognostic group with respect to recurrence-free survival. Importantly, phospho-ERK levels were of predictive significance only in high-risk patients, who received adjuvant (postoperative) chemotherapy, but not in high-risk patients not receiving such therapy. Our results suggest that high cancer cell levels of phospho-ERK predict poor responsiveness to both preoperative and postoperative chemotherapy of rectal cancer.

## INTRODUCTION

Treatment of rectal cancer has been substantially improved by the introduction of total mesorectal excision surgery but the disease is still associated with considerable morbidity and mortality. Patients with locally advanced rectal adenocarcinomas (LARC) are commonly offered neoadjuvant treatment with radiochemotherapy (RCT) prior to surgery [1–4]. After surgery, adjuvant chemotherapy may be administered to high-risk patients. However, it has been questioned whether all such patients benefit from adjuvant therapy and there is a paucity of reliable markers to predict the response [5].

ERK 1 and 2 (extracellular signal-regulated kinases 1 and 2, also known as mitogen activated protein kinases: MAPK p44 and p42) regulate cell division, apoptosis and motility [6, 7]. Although encoded for by different genes, they subserve identical, redundant functions. ERKs are activated by an array of upstream regulators, including the EGF receptor (EGFR), RAS, and RAF. Activating mutations in genes encoding these regulators are central to the development and/or progression of colorectal and other cancers [8–10]. ERK activation proceeds through a cascade that is initiated by binding of EGF (or other growth factors) to cell surface receptors. Receptor binding initiates conversion of RAS to its active, GTP-bound form. Activated RAS starts a chain of events culminating in the activation of RAF, which, in turn, activates the MAPK/ERK kinases (MEK) 1 and 2. MEKs activate ERKs by dual phosphorylation to phospho-ERKs. Most phospho-ERKs translocate to the nucleus, where they stimulate transcription of genes regulating cell cycle progression and apoptosis [6, 7, 10–12]. MAPK phosphatases (MKPs, also referred to as dual specificity phosphatases; DUSPs) remove the activating phosphorylations on both threonines and tyrosines in phospho-ERKs and, thus, terminate their activity. Of ERK-targeting MKPs, class I enzymes (MKP-1/DUSP1, DUSP2/PAC-1, MKP-2/DUSP 4 and DUSP5) are nuclear and class II enzymes (MKP-3/DUSP6, MKP-X/DUSP7 and MKP-4/DUSP9) are cytoplasmic. MKP-1 expression is stimulated by pERKs, which, hence, participate in a negative feedback loop. The expression of such phosphatases has been linked to cancer progression and therapy responses [7]. Thus, steady-state levels of phospho-ERKs are dictated by both activating and inactivating regulators.

*In vitro* studies have demonstrated that MEK inhibitors (which abolish ERK phosphorylation) sensitize cultured cancer cells to 5-fluorouracil (5FU) as well as to radiation [13–15]. Using diagnostic (pre-treatment) biopsies, we have previously demonstrated that high phospho-ERK levels in rectal cancer cells are associated with poor RCT responses in terms of tumor regression and downstaging [10]. We now report that high cancer cell levels of phospho-ERK also subdivide high-risk patients into a favourable and less favourable group with respect to recurrence-free survival (RFS). This effect is highly significant for high-risk patients receiving postoperative chemotherapy, but not for high-risk patients, who do not receive such therapy. These results suggest that high phospho-ERK levels in cancer cell predict poor responses both to neoadjuvant and adjuvant chemotherapy.

## RESULTS

**Figure 1:**
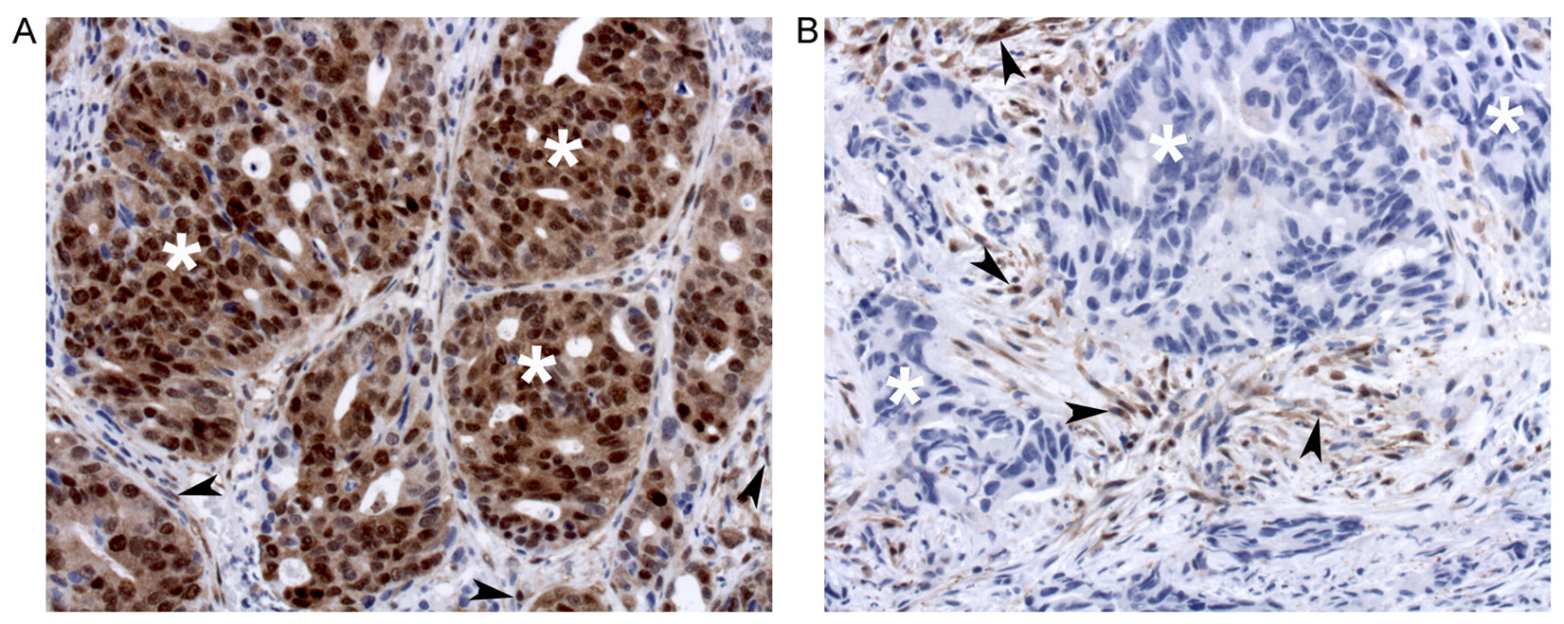
Phospho-ERK staining of tumors that show strong (**A**) or no (**B**) staining of cancer cells (exemplified with white asterisks). Note that stromal cells (arrowheads) are strongly stained in both A and B.

Pre-treatment biopsies stained for phospho-ERK showed variable degrees of reactivity in cancer cells as well as in stromal and inflammatory cells (Figure 1A and 1B). Staining for phospho-ERK was most intense in the nuclei and weaker in the cytoplasm of all immunopositive cells (Figure 1A and 1B). This concurs with the fact that most ERK is rapidly imported into the nucleus following its phosphorylation [6, 7]. Controls (type-matched IgG1 as well as lambda phosphatase pre-treatment) were negative. Staining of stromal and/or inflammatory cells was as strong in biopsies that contained positive cancer cells (Figure 1A) as in biopsies that contained weakly reactive or no positive cancer cells (Figure 1B). This internal control attested that the quality of tissue fixation and staining was optimal in all biopsies. Finally, staining for ERK protein (irrespective of phosphorylation status) showed that it was present also in cancer cells that showed no phospho-ERK staining. Accordingly, as previously noted for colorectal carcinomas [11], lack of ERK phosphorylation did not reflect lack of ERK protein expression.

Two observers, who were unaware of the clinical data and outcomes, scored all (coded) specimens independently. Cancer cell nuclei, as well as nuclei of intertwining stromal cells, were scored for phospho-ERK staining with respect to average intensity (on a scale from 0–3 with 0 representing no staining and 3 intense staining) and number (in 10% increments using a scale from 0–10). Multiplication of the intensity and number scores produced observed ranges of product scores from 0–21.5 in cancer cell nuclei (Supplementary Figure 1) and of 1–27.0 in stromal cell nuclei (theoretical range: 0–30 for both). There was excellent agreement between the two observers (kappa = 0.76) and results are presented as averages of their scores. The product score showed no significant correlation to baseline clinical data (age, gender, and, as assessed by MRI; tumor length, tumor location, distance from the mesorectal fascia, cN or cT).

Initially, we validated the scores by determining whether we could reproduce the effects of our previous finding [10] that pre-treatment phospho-ERK scores could predict effects of RCT on downstaging and tumor regression grade (TRG) in this new cohort of patients.

**Figure 2:**
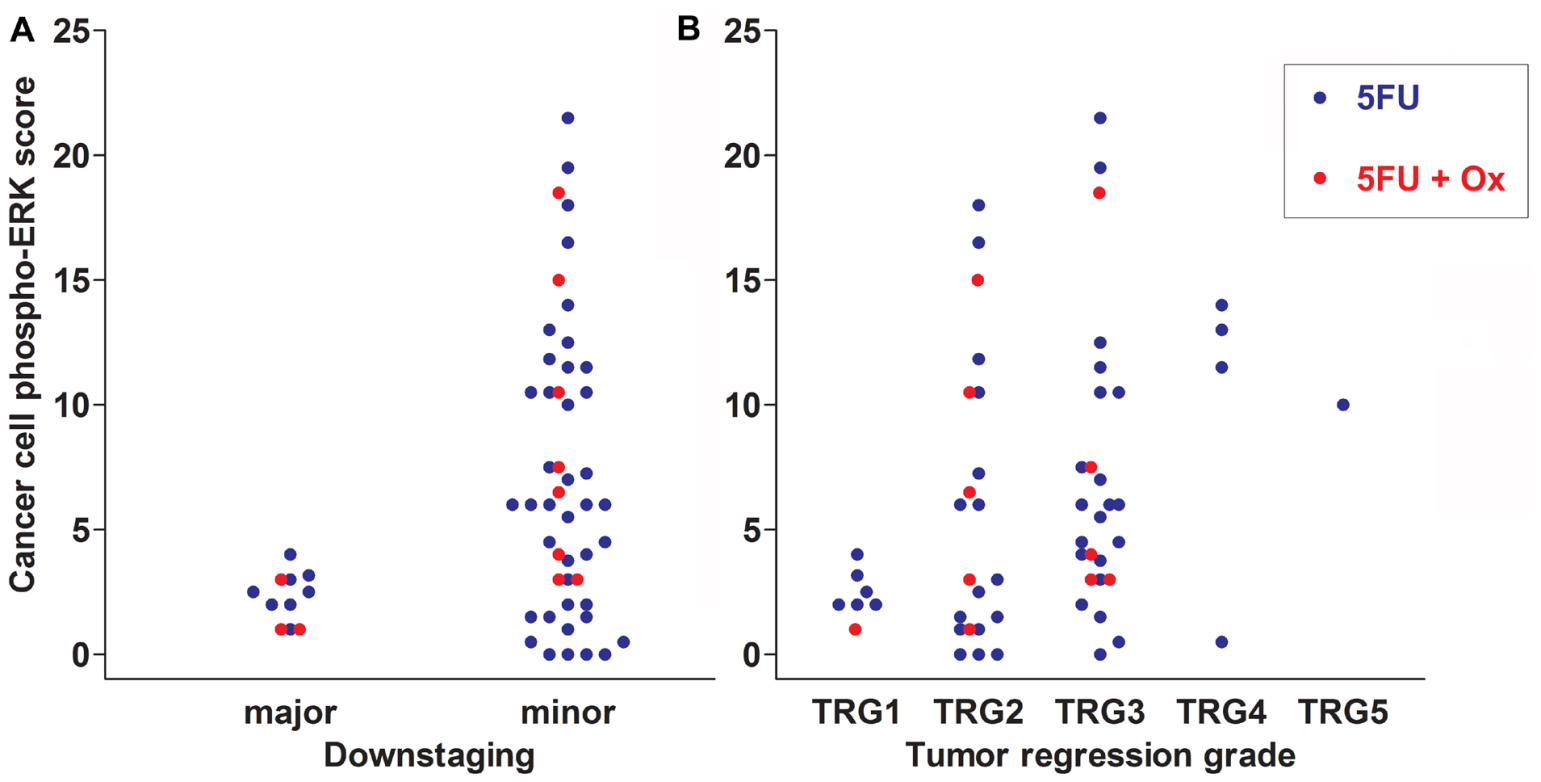
Scatter plots showing downstaging (**A**) and TRG scores (**B**) in relation to cancer cell phospho-ERK scores for all 59 patients of cohort 1. Data for patients treated with 5FU alone are shown as blue dots and for 5FU+oxaliplatin as red dots. Note that none of the patients with cancer cell phospho-ERK levels above the median score (4.5) respond with either major downstaging or complete tumor regression (TRG1 in A). The difference in cancer cell phospho-ERK scores between major and minor downstaging is significant both for all patients (*p* = 0.007) as well as for the 5FU+oxaliplatin group (*p* = 0.02). It is borderline significant (*p* = 0.06) for the group receiving 5FU alone. Note in B that the cancer cell phospho-ERK score is lower in patients experiencing higher degrees of tumor regression (1 = total; 5 = none). The difference in median cancer cell phospho-ERK scores between the TRG1 and TRG3-5 group is significant for all patients (*p* < 0.02) as well as for the 5FU group alone (*p* = 0.03), whereas the 5FU+oxaliplatin group only contained a single case of complete tumor regression.

**Figure 3:**
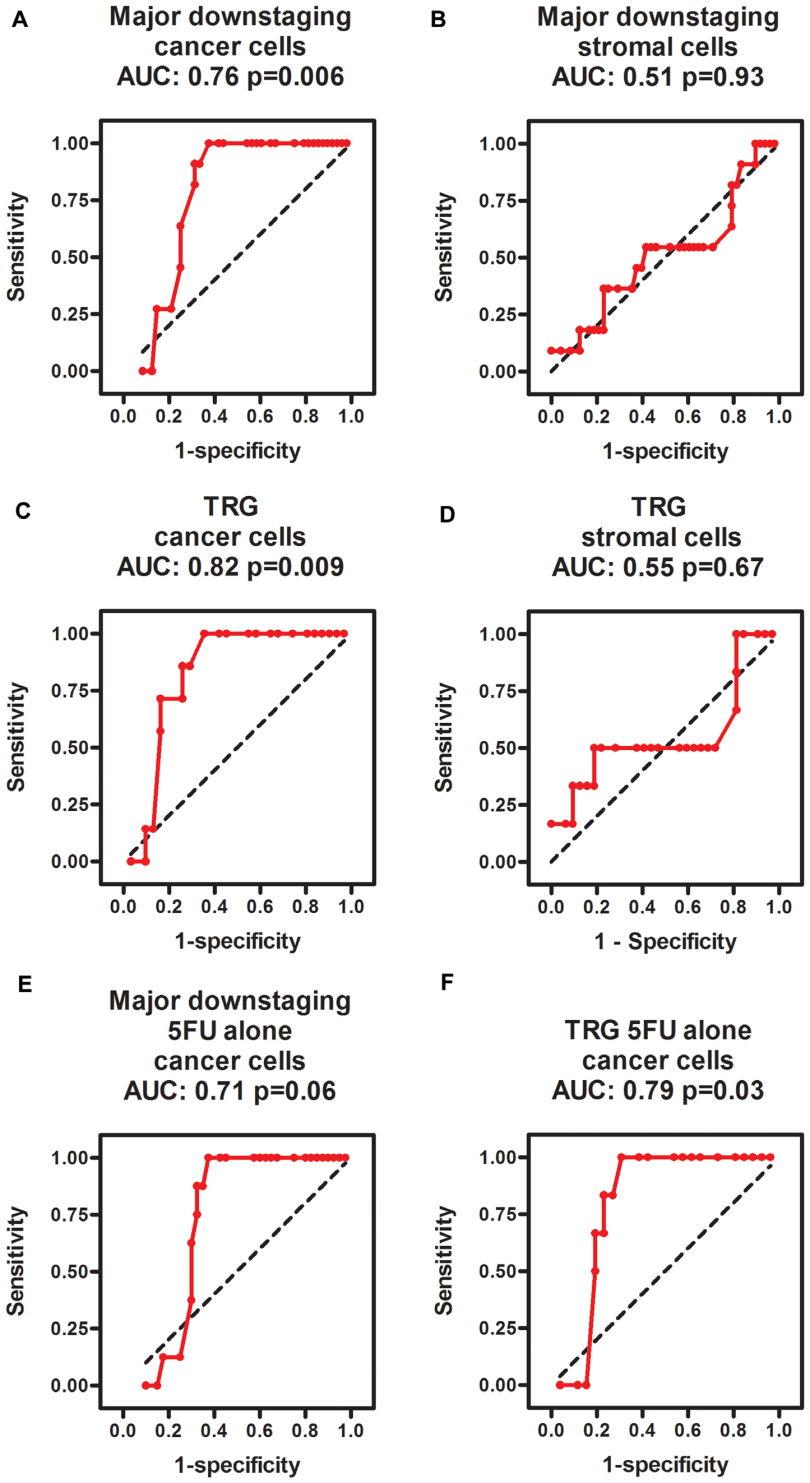
ROC diagrams showing the predictive power of phospho-ERK scores in cancer cells (**A**, **C**, **E**, **F**) but not in intertwining stromal cells (**B**, **D**) for predicting major downstaging (A, B, E) and TRG1 (C, D, E, F) in all patients (A–D) as well as the subgroup of patients treated with 5FU alone (E, F). The black dotted line in each diagram illustrates an imaginary line of no predictive power (AUC = 0.5).

Major downstaging was defined as cT-ypT>1, ypN=0 (all major downstagers were without nodal involvement). Patients with cancer phospho-ERK scores above the median (≥4.5) never achieved major downstaging (Figure 2A). Moreover, no patients with scores below 4.5 showed a ypT category above 2 following RCT. Notably, however, whilst a score above the median predicted poor downstaging, a score below the median was no guarantee for major downstaging (Figure 2A). The median cancer cell phospho-ERK score was significantly lower (Mann-Whitney *U* -test *p* = 0.0068) for major downstagers (2.5; *n* = 11) than that for patients showing poor or no downstaging (6.0; *n* = 48). ROC analyses (Figure 3A) produced an area under curve (AUC) of 0.76 (*p* = 0.006). In contrast, phospho-ERK scores of stromal cell nuclei in the same tumor sections showed no predictive power (Figure 3B).

Similarly, no patients with cancer cell scores above the median (4.5) achieved total tumor regression (TRG1; Figure 2B) and the median cancer cell phospho-ERK score was significantly (*p* = 0.02) lower for TRG1 responders (2.25) than for patients who responded moderately to poorly (TRG3-5; 6.0). All patients achieving TRG1 were devoid of lymph node metastases in the resection specimens (pN=0) and, thus, represented complete pathological responses (cPRs). Whilst a high phospho-ERK score was incompatible with TRG1/cPR, a low score did not necessarily indicate a good TRG response (Figure 2B). ROC analyses (Figure 3C) for separating total responders (TRG1) from poor responders (TRG3-5) showed an AUC of 0.82 (*p* = 0.009), whilst phospho-ERK scores of stromal cell nuclei in the same tumor section showed no predictive power (Figure 3D). These results pertained to all patients. We additionally analyzed the subset of patients who received 5FU alone. The results showed that the effects of pERK scores on downstaging and TRG could be reproduced in this subset of patients (Figure 3E and 3F). Patients receiving combined 5FU+Oxaliplatin treatment were too few for statistical analyses but the same trend was observed (Figure 2A and 2B shows color-coded dots for both treatments). These data show that our previously reported results [10] could be reproduced with a high degree of fidelity in a second patient cohort.

**Figure 4:**
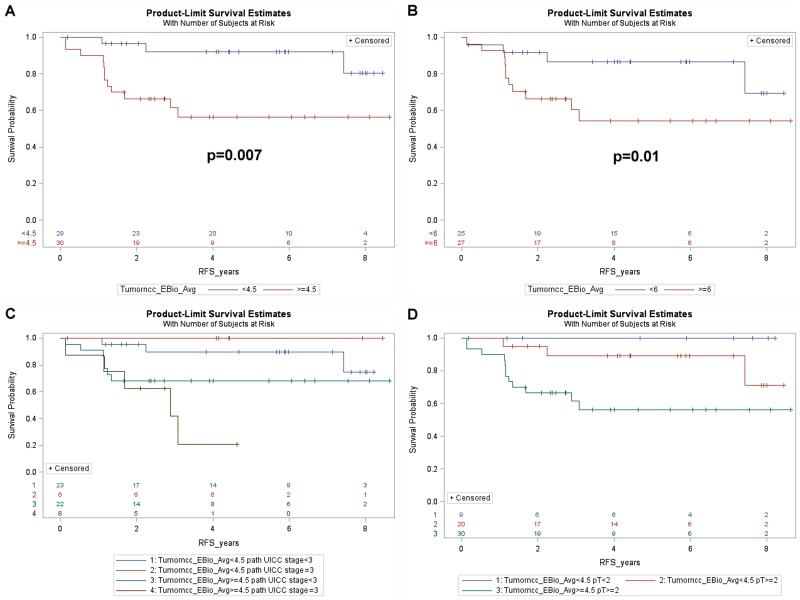
Kaplan-Meier plots showing (**A**) recurrence free survival of all patients dichotomized by the median cancer cell phospho-ERK score (4.5). Note that patients with a score above the median show poorer RFS. (**B**) RFS for patients with UICC grade ≥ II in relation to the median cancer cell phospho-ERK score. Note that the median (6.0) is higher for these patients than for the entire patient group. Also note that patients with lower cancer cell phospho-ERK scores show significantly better (*p* = 0.01) RFS than patients with higher scores. (**C**) RFS for all 59 patients subdivided by the median cancer cell phospho-ERK score as well as by UICC grade 3. Note that patients with low cancer cell phospho-ERK scores show equally good survival, irrespective of the TNM grade, whereas patients with high cancer cell phospho-ERK scores and high grade show dismal RFS. (**D**) RFS in relation to T classification (ypT) and the median cancer cell phospho-ERK score for all patients. Note that patients with a ypT of 2 or more show almost as good RFS as ypT0-1 patients provided they have a low cancer cell phospho-ERK score, whereas such patients with a high score show much poorer RFS. Also note that, in keeping with the effects of the cancer cell phospho-ERK score on downstaging, there are no patients with a high cancer cell phospho-ERK score and a ypT below 2.

We next examined whether the cancer or stromal nuclear phospho-ERK scores had any impact on RFS. It emerged that patients with cancer phospho-ERK levels above the median (≥4.5) had significantly shorter RFS than patients with phospho-ERK levels below the median (5-year-RFS: 56.3% versus 92.0%, *p* = 0.007) (Figure 4A). When used as a continuous variable, the cancer pERK score emerged as a borderline significant predictor for RFS (*p* = 0.07). In contrast, stromal phospho-ERK scores were not predictive for RFS, whether used as a continuous variable (*p* = 0.69; HR: 0.98, 95% CI 0.89–1.10) or dichotomized by the median (*p* = 0.7).

Further analyses revealed that the predictive value of the cancer cell phospho-ERK scores mainly reflected an impact on high-risk patients. The impact of phospho-ERK levels on RFS in patients with UICC stage II or III was significant at the *p* = 0.01 level (Figure 4B). Note that the median score for cancer cell phospho-ERK (6.0) in stage II-III patients was higher than the population median (4.5). It was noteworthy that UICC stage III patients, who had low phospho-ERK scores, showed an RFS that was not significantly different from that of stage I-II patients (with low phospho-ERK scores) whilst stage III patients with high phospho-ERK scores showed very poor RFS (Figure 4C). These differences were evident also when only T categories were analysed (Figure 4D).

**Figure 5:**
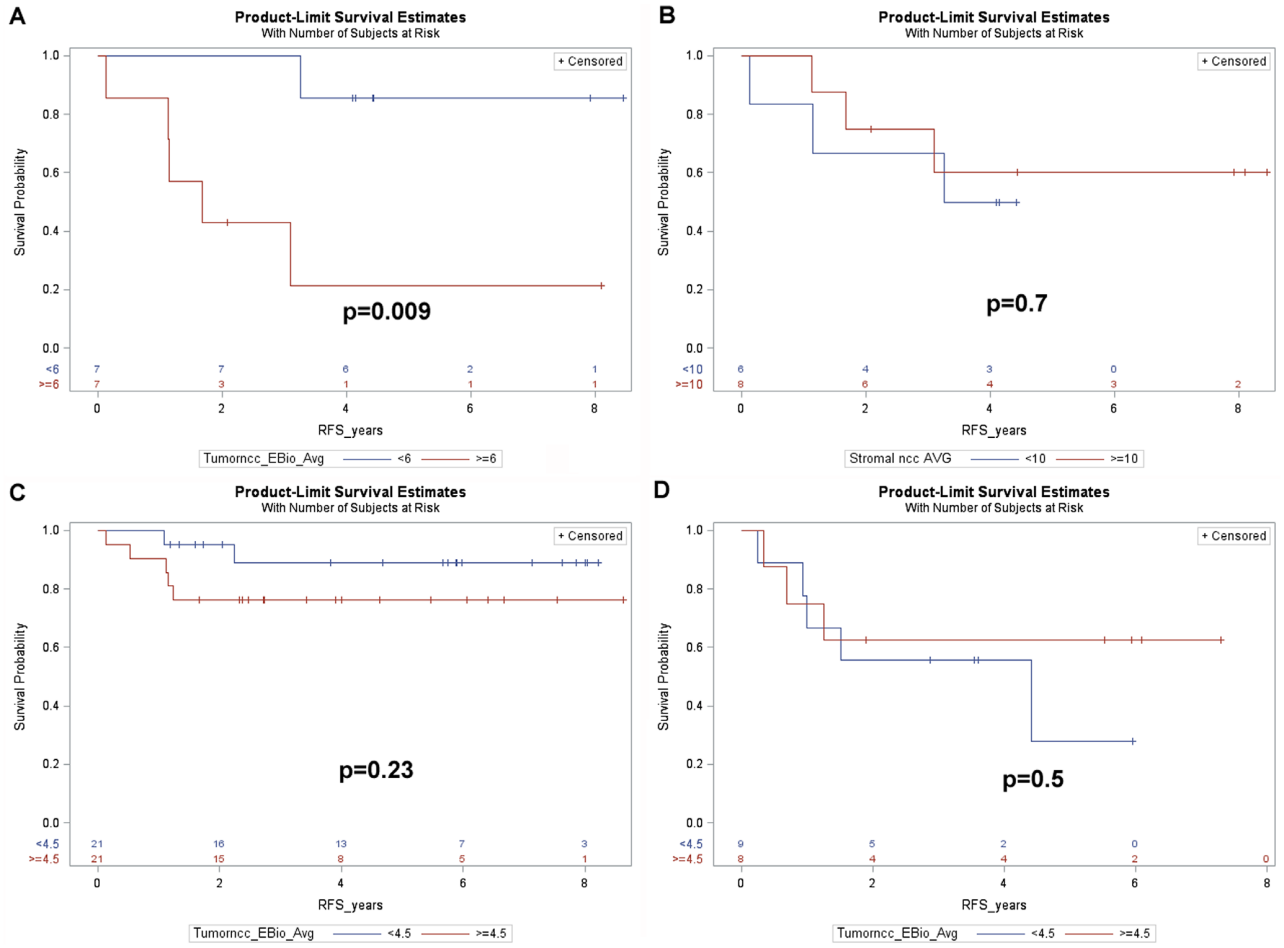
Kaplan-Meier plots showing (**A**) RFS for the 14 patients of cohort1, who received adjuvant treatment, dichotomized by the median cancer cell phospho-ERK score (6.0) (**B**) RFS for the 14 patients of cohort 1, dichotomized by the median stromal cell phospho-ERK score. (**C**) RFS for the 42 remaining patients of cohort 1, who did not receive adjuvant treatment (3 further patients of cohort1 lacked data regarding adjuvant treatment) dichotomized as in A. (**D**) RFS for the 17 patients of cohort 2, who fulfilled the criteria, but who did not receive, adjuvant treatment, dichotomized by the median cancer cell phospho-ERK score.

**Figure 6:**
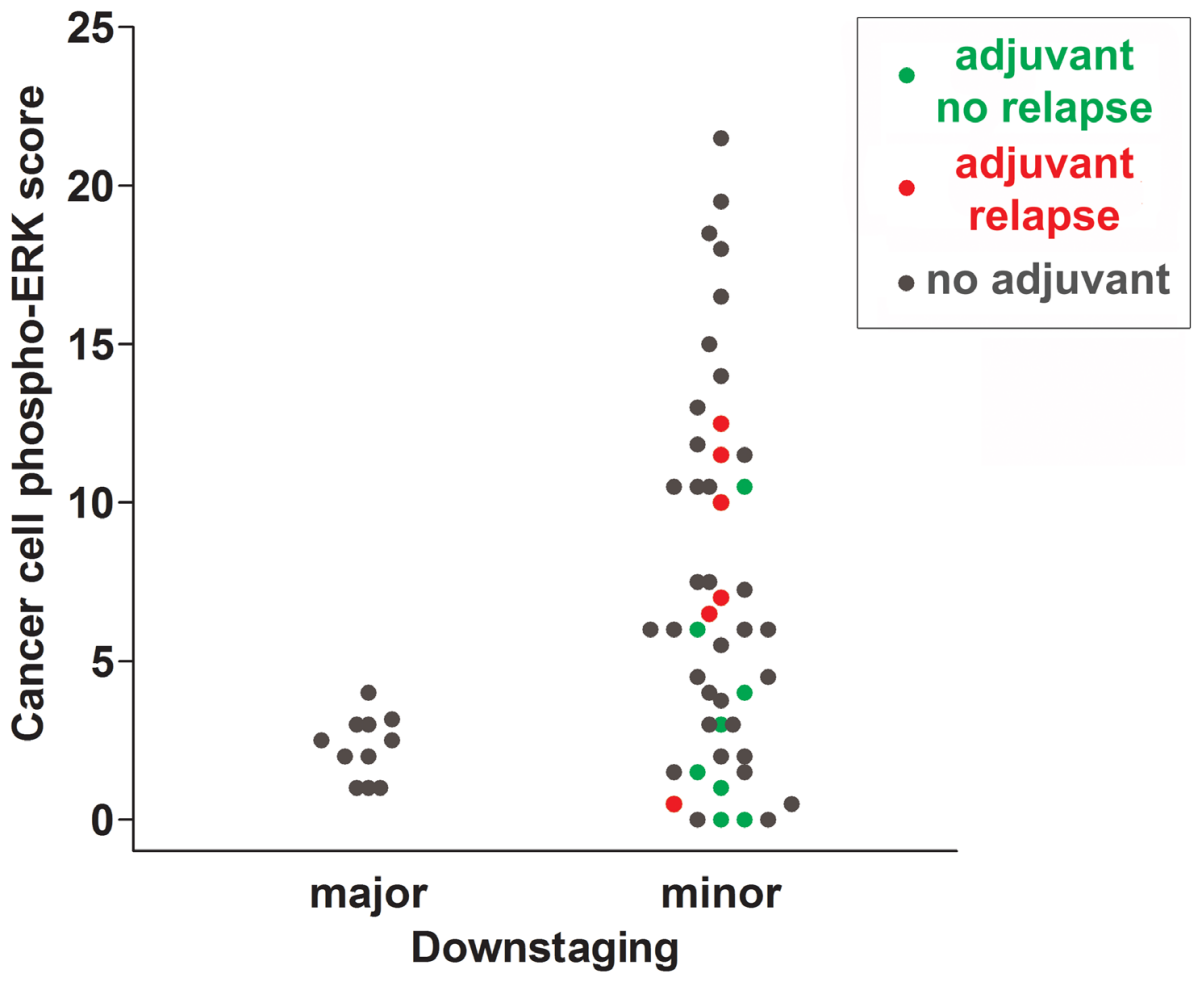
Scattergram summarizing cancer cell phospho-ERK scores for all individuals of cohort 1 Patients receiving adjuvant treatment without subsequent recurrences are indicated with green dots and patients receiving adjuvant treatment with subsequent recurrences are indicated with red dots. Patients not receiving adjuvant treatment are shown in grey. Note that the majority of patients not experiencing recurrences had phospho-ERK scores below the median.

Since patients with poor prognostic indicators frequently are offered postoperative (adjuvant) chemotherapy, we examined whether these results could be explained by effects of phospho-ERK levels on the outcome of such therapy. Indeed, the positive prognostic impact of low cancer cell phospho-ERK levels mainly, if not exclusively, reflected effects in the subset of patients who received adjuvant therapy (5-year-RFS: high score: 21.4% versus low score: 85.7%, *p* = 0.009) (Figure 5A). This group consisted of 14 patients (Table 1), including 12 UICC stage III, 2 UICC stage II with a CRM distance <1 mm and one UICC stage I with non-radical surgery. There were no differences in adjuvant treatment between patients having tumors showing high and low pERK scores: 5 in each group had received combined 5FU+oxaliplatin whilst the remaining two in each group had received 5FU alone. The phospho-ERK scores of stromal cells showed no predictive power (5-year-RFS: high score: 60%; low score: 50%; *p* = 0.7) (Figure 5B). The cancer phospho-ERK score also emerged as significant when used as a continuous variable (HR: 1.32; 95% CI: 1.04–1.67; *p* = 0.025). Of other variables considered to be important to postoperative disease progression (ypN, ypT, UICC stage, critical CRM distance, tumor location, perineural and intravascular growth), only CRM (at <1 mm) was borderline significant (HR: 0.22; 95% CI: 0.22–1.11; *p* = 0.07). In multivariate analysis, the (continuous) pERK score remained significant (HR: 1.38; 95% CI: 1.04–1.84; *p* = 0.027) whilst CRM<1 mm remained borderline (HR: 0.163; 95% CI: 0.025–1.049; *p* = 0.06) and none of the remaining variables emerged as significant. Microsatellite instability was detected in only one of 11 patients examined (by immunohistochemistry for mismatch repair proteins MSH2, MSH6, MLH1 and PMS2, cf ref 11). It was there not included in the multivariate analysis but, following exclusion of the single MSI tumor from the data set, cancer cell phospho-ERK levels remained significant (HR: 1.305; 95% CI: 1.02–1.67; *p* = 0.036). Table 1 illustrates the characteristics of the 14 patients. Apart from the differences in median pERK scores, the two groups are remarkably similar and, as can be seen from the Kaplan-Meyer curve (Figure 5A), only one patient with a low cancer phospho-ERK score experienced a (late) relapse. However, there was a small difference indicating that patients who responded better to RCT (with respect to TRG and downstaging) also responded better to adjuvant therapy to the preceding RCT (Table 1). This difference was not significant, probably reflecting that good RCT responders did not exist in the adjuvant-treated group (Figure 6).

**Table 1:** Characteristics of the 14 patients of cohort 1, who received adjuvant therapy, subdivided according to relapses

Variable	No recurrences (*n* = 8)	Recurrences (*n* = 6)^1^
**age**	67.5 (48–76) years	66.5 (55–69) years
**gender (female: male ratio)**	3:5	0:6
**cancer cell phospho-ERK score**	2.3 (0–10.5)	8.6 (0.5–12.5)
**stromal cell phospho-ERK score**	10.0 (1.5–27.0)	10.3 (5.0–14.8)
**TRG**	2.5 (2–3)^2^	3.0 (2–5)^3^
**downstaging (cT-ypT)**	0 (0–1)	0 (–1^4^–1)
**pT**	3 (2–3)	3 (2–4)
**pN**	1 (0–2)	1 (0–2)
**deaths (ratio)**	0:8	2:6

If not otherwise indicated, numbers refer to medians with total ranges given in brackets.

^1^ 3 patients had liver metastases, 2 had lung metastases and one had disseminated disease.

^2^ 4 TRG2 and 4TRG3.

^3^ 2 TRG2, 3TRG3, 1 TRG5.

^4^ one patient upstaged during RCT.

Forty-two of 56 cohort 1 patients did not receive adjuvant therapy (adjuvant therapy data were lacking for 3 of the 59 patients; Table 2). In the patients not receiving adjuvant treatment, the cancer phospho-ERK score did not emerge as a significant predictor for RFS (5-year-RFS: high score: 76.2%; low score: 88.9%; *p* = 0.23) although a weak trend in favour of low scores was noted (Figure 5C). These results show that the positive effect of a low pERK score on RFS of all patients of cohort 1 mainly reflected effects on the 14 patients, who did receive adjuvant therapy. However, the 42 patients, who did not receive adjuvant therapy, represented, of course, low-risk patients. We therefore included cohort 2, encompassing 17 patients having UICC stage III and/or a CRM distance below 1 mm, which, according to DCCG criteria, were candidates for adjuvant therapy. These patients had not received any adjuvant treatment because of previous drug toxicity and/or comorbidities that had resulted in reductions or discontinuation of their neoadjuvant RCT (which is why they had not been included in cohort 1 for studying downstaging and TRG). In this “control” group, the pERK score, as dichotomized by the median, showed no significant predictive power (5-year-RFS: high score: 62.5%; low score: 55.6%; *p* = 0.50) (Figure 5D).

**Table 2:** Characteristics (median and ranges, where relevant) for patients receiving full preoperative radiochemotherapy (RCT)

	Full RCT
**n (gender)**	59 (15 f, 44 m)
**age**	67 (43–84)
**full image-gudied RT**	59
**RCT with 5FU**	48
**RCT with 5FU+oxaliplatin**	11
**postop chemotherapy**	14 (*n =* 56)
**cT2**	2 (*n =* 59)
**cT3**	45 (*n =* 59)
**cT4**	12 (*n =* 59)
**cN0**	12 (*n =* 47)
**cN1**	18 (*n =* 47)
**cN2**	17 (*n =* 47)
**tumor length (cm)**	6 (2–10; *n =* 21)
**mesorectal fascia distance (mm)**	1.5 (0–15; *n =* 26)
**low/medium-high tumors**	16/22 (*n =* 38)

MRI was used for recording clinical tumor (cT) and lymph node classification (cN), median tumor length, median distance from the mesorectal fascia (when applicable) and tumor location. Numbers given in parentheses indicate number of records available for each variable.

## DISCUSSION

The most important new finding of our study is that the cancer, but not stromal, phospho-ERK score, as determined on diagnostic biopsies, constitutes a significant predictor for RFS of LARC patients. We were able to demonstrate that this effect mainly, if not exclusively, reflected effects of the response to adjuvant CT administered to high-risk patients following surgery. Thus, although our data fully confirms that the same phospho-ERK scores predict the response to preoperative RCT [10], the present data extends these findings by showing effects on the outcome of adjuvant therapy. There were only a small difference between the RCT response in terms of either TRG or downstaging and the subsequent response to adjuvant therapy. This may reflect the fact that all patients who received adjuvant therapy were (almost by definition) minor (cT-ypT≤1) downstagers and showed incomplete TRG responses (Table 1). However, as indicated in Table 1, there was a trend suggesting that a better, albeit small, response to RCT correlated to a better response to adjuvant therapy. It is worthy of note that whilst a high cancer (but not stromal) phospho-ERK score never was associated with major downstaging or total tumor regression, the reverse was not true. Thus, a low cancer phospho-ERK score did not serve to exclude a poor response with respect to these outcomes. However, most of these patients showed best response to subsequent adjuvant therapy (as illustrated by color-coding in Figure 6).

Our results are consonant with previous studies showing that high levels of phospho-ERK interferes with effects of 5FU as well as of radiation on cancer cells *in vitro* [13–15]. Together, the results support the idea that high phospho-ERK levels render rectal cancer cells insensitive to CT. This could be due to multiple factors. Firstly, both chemotherapy and radiation work by inducing apoptosis and phospho-ERK constitutes an important regulator of this event [6, 7]. Additionally, phospho-ERK stimulates expression of the anti-apoptotic protein survivin [16, 17] which correlates with survival of RCT-treated colorectal cancers [18]. This further underlines the importance of cancer cell phospho-ERK levels for responses to RCT. A low cancer phospho-ERK score was not invariably associated with a good RCT response but was, as pointed out above, statistically associated with a better RFS in patients receiving adjuvant therapy. This is most likely due to additional factors, which determine the sensitivity of tumor cells to 5FU. Such factors include drug transporters, microRNA expression patterns and several others. Accordingly, we posit that the impact of low phospho-ERK levels may be cancelled by such factors, whilst the apoptosis-preventing effect of high phospho-ERK levels is dominant.

Separate analysis of the 42 patients of cohort 1, who did not receive adjuvant therapy, showed no significant effect of cancer phospho-ERK scores on RFS. Thus, although we observed that phospho-ERK scores predicted significantly better RFS for the patients of cohort 1 (and could predict much better RFS for the high-risk patient subgroup) this mainly reflected effects on the group of the 14 patients who did receive adjuvant treatment. To determine whether this was an effect relating directly to interference of high phospho-ERK with CT we therefore studied an additional group of 17 patients, who were eligible to, but who did not receive, adjuvant therapy (due to previously experienced drug toxicity or comorbidities). These patients did not show any significant effect of cancer phospho-ERK levels on RFS. We take this as a strong indication that the phospho-ERK score predominately predicts the CT response. However, it is highly likely that phospho-ERK also affects many other aspects of cancer cell growth, survival and motility and, indeed, in the 42 non-adjuvant receiving patients, a non-significant trend towards better RFS for low cancer phospho-ERK scores was observed.

It is of considerable interest that the phospho-ERK scores could be used to stratify high-risk patients (like UICC stage III) into a poor and a good prognostic group. Although, again, this mainly (or exclusively) reflected effects on adjuvant therapy it is remarkable that levels determined in diagnostic, pre-treatment biopsies have so strong an impact subsequent to both neoadjuvant and adjuvant treatment. It is a limitation of the present study that the number of patients receiving postoperative adjuvant treatment is relatively small. Nevertheless, the difference in RFS between the high and low phospho-ERK group is very marked and highly significant. A follow-up study addressing a larger patient material is currently being planned. If these findings can be confirmed in an independent material it opens for use of phospho-ERK as a new predictive marker that perhaps may be useful also in other cancer forms, like colonic carcinoma. It also poses the question whether MEK inhibitors, which increase the efficiency of 5FU in cultured cells [15], could be useful supplements to neoadjuvant/adjuvant therapy of rectal carcinoma patients. Such inhibitors are now in clinical use and do also target oncogenic MEK mutations in colorectal cancers, which may occur secondarily to BRAF inhibitor treatment [21].

## MATERIALS AND METHODS

### Ethics statement

The study was approved by the Danish Data Protection Agency (AHH-2016-044: I-Suite 04853) and Ethical Committee (protocol H-15015151).

### Patients

Fifty-nine patients, consecutively diagnosed with LARC at three major Copenhagen hospitals (Herlev, Hilleroed and Gentofte) during 2008–2015, received full concomitant neoadjuvant RCT, including image-guided RT and had no distant metastases at the time of diagnosis and no coexisting malignancies (cohort 1). This patient cohort did not encompass any of the patients reported in our previous study [10]. Patient details are summarized in Table 2. The majority of these patients (*n* = 48) received peroral 5-fluorouracil (5FU) alone whilst 11 patients received 5FU in combination with oxaliplatin. Subsequently, 14 patients received postoperative (adjuvant) chemotherapy (CT) (Table 1). Ten of these patients received adjuvant therapy with 5FU combined with oxaliplatin, whereas 4 patients received 5FU alone. Tumor location was noted as low (starting 0–5 cm above the anal verge), medium (5–10 cm) and high (10–15 cm). Tumor length, distance from the mesorectal fascia, clinical tumor (cT) and node (cN) classifications were determined by magnetic resonance imaging (MRI). Tumor length was used for indicating tumor size.

We additionally analysed data from 17 patients (cohort 2), who also had been diagnosed with LARC during the same time-span at the same hospitals as cohort 1. However, these patients were not included in cohort 1, because they had not received full neoadjuvant CT treatment (due to drug toxicity or comorbidities). Postoperatively, these patients fulfilled the Danish colorectal cancer group (DCCG) criteria for adjuvant (UICC stage III and/or a CRM distance below 1 mm) therapy but they did not receive such therapy because of the above concerns regarding toxicity and/or comorbidities. Specimens from both cohort 1 and 2 were simultaneously stained, blind coded and scored in the same run (as detailed in the subsequent sections).

### Tissue analyses

Endoscopic biopsies were immediately fixed in 10% buffered neutral formalin and embedded in paraffin. Adjacent 3-μm sections were stained with HE and for immunohistochemistry. The latter involved demasking of sections at high pH and staining with two previously well-characterized [10, 11] mouse monoclonal antibodies, specifically detecting phosphorylation of Thr202/Tyr204 in ERK1 and of Thr185/Tyr187 in ERK2(clone Milan8R; mouse IgG1, eBioscience/Affymetrix, San Diego, CA), or detecting ERK1/2 protein regardless of its phosphorylation status (clone L34F12; mouse IgG1, #4696, Cell Signaling Technology, Leiden, Netherlands), using automated staining machines and immunoperoxidase detection as described [10–12]. Controls included substitution of the monoclonal antibodies with type-matched control mouse IgG1 as well as dephosphorylation prior to phospho-ERK staining, as described [10, 11]. Validation of the phospho-ERK antibody included, besides the controls outlined above, full length Western blotting, which revealed only the expected 42- (phospho-ERK2) and 44-kDa (phospho-ERK1) bands [11]. Sections from surgical resection specimens were stained with HE for determining TRG, using the Mandard scale [19, 20] from 1 (total regression) to 5 (no regression). This scale was routinely used in the hospitals involved.

### Scoring and statistics

Two observers independently scored all sections stained for phospho-ERK from all patients (cohort 1+2) in the same run using blind-coded specimens. The average staining intensities of cancer cell nuclei in each section were graded as absent (0), weak (1), moderate (2) or strong (3) and percentages of stained cancer cell nuclei were scored as 0 (0), 1–10 (1), 11–20 (2), etc. in 10%-increments. RFS, in days counted from the day of primary surgery, were analysed. All relapses involved metastatic disease. Downstaging was defined as the difference (cT-ypT) between T categories, as determined by MRI (cT) before RCT and by pathology (ypT) following RCT and surgery. Statistics used SAS (version 9.4, SAS Institute, Cary, NC). Survival curves were constructed with the Kaplan-Meier method and equality over strata evaluated with log-rank statistics. The Cox proportional hazard model was used for multivariate analysis and included estimates of the hazard ratio (HR) with 95% Wald confidence limits. Multivariate analysis included stepwise elimination of variables. Differences in TRG and downstaging in relation to phospho-ERK scores were analysed by receiver operating curves (ROCs) as described [10]. The significance level was set at 5%.

## SUPPLEMENTARY MATERIALS FIGURES AND TABLES



## Abbreviations

5FU: 5-fluorouracil
AUC: area under curve
CI: 95%confidence interval
cN: clinical lymph node classification
cPR: complete pathological response
CRM: circumferential resection margin
cT: clinical T classification
CT: chemotherapy
DCCG: Danish colorectal cancer group
EGFR: EGF receptor
ERK: extracellular signal-regulated kinase
HR: hazard ratio
LARC: locally advanced rectal adenocarcinomas
MAPK: mitogen activated protein kinase
MEK: MAPK/ERK kinase
MRI: magnetic resonance imaging
phospho-ERK: active, dually phosphorylated ERK 1/2
pN: pathological lymph node classification (after RCT: ypN)
ypT: pathological tumor classification (after RCT)
RCT: radiochemotherapy
ROC: receiver operating curve
RFS: recurrence-free survival
TRG: tumor regression grade (0 = total and 5 = no regression)
UICC: Union for International Cancer Control.
