# Supplementary material for: Phospho-ERK levels as predictors for chemotherapy of rectal carcinoma
Source: Oncotarget. 2019 Mar 1;10(18):1745–55. doi: 10.18632/oncotarget.26741 (PMC6422203; doi:10.18632/oncotarget.26741)
Supplement: Supplementary file 1 [file oncotarget-10-1745-s001.pdf]

Phospho-ERK levels as predictors for chemotherapy of rectal carcinoma

SUPPLEMENTARY MATERIALS

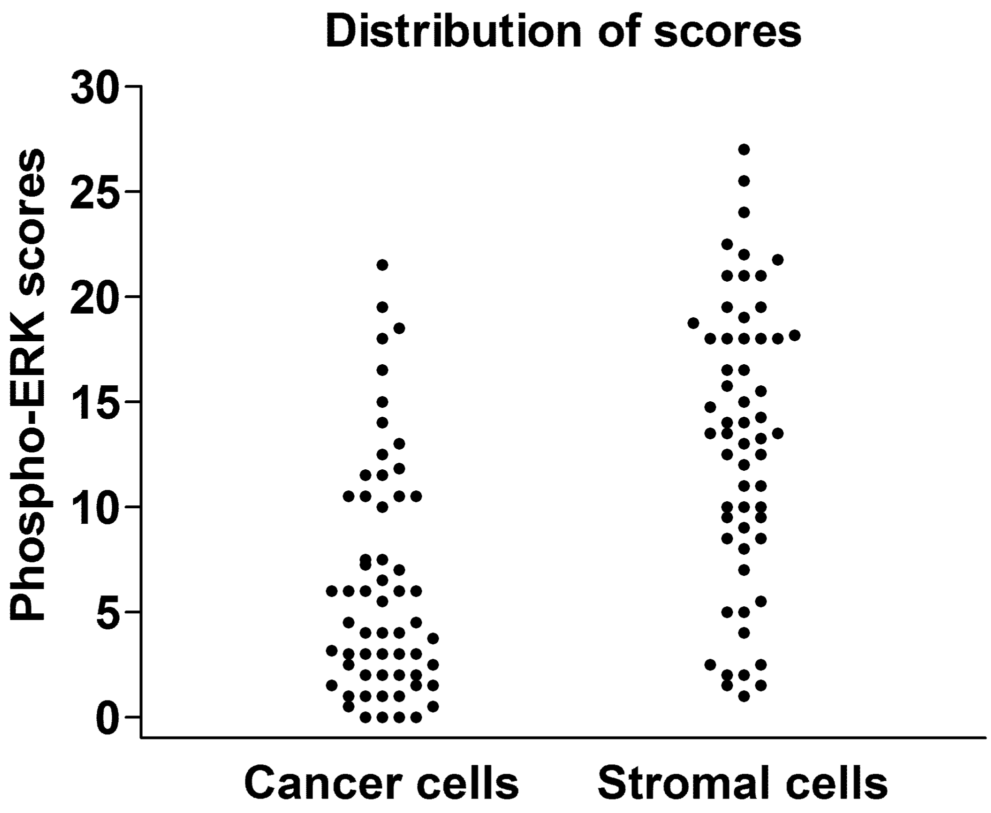

Supplementary Figure 1: Scattergram illustrating the distribution of scores for nuclei in cancer cells and stromal cells.
